# Supplementary material for: BCEPS: A Web Server to Predict Linear B Cell Epitopes with Enhanced Immunogenicity and Cross-Reactivity
Source: Cells. 2021 Oct 14;10(10):2744. doi: 10.3390/cells10102744 (PMC8534968; doi:10.3390/cells10102744)
Supplement: Supplementary file 1 [file cells-10-02744-s001.zip › Table_S1.pdf]

Table S1: Genetic frequency of HLA-DRB1 alleles in 4 distinct ethnic groups in USA

HLA-DRB1	White	Black	Asian	N.A. Natives	HLA-DRB1	White	Black	Asian	N. A. Natives
DRB1*01:01	0.085	0.026	0.0274	0.07	DRB1*11:17	0	0.0002	0	0
DRB1*01:02	0.014	0.0399	0.0003	0.01	DRB1*12:01	0.017	0.0395	0.0289	0.013
DRB1*01:03	0.009	0.0023	0.0003	0.01	DRB1*12:02	0.0003	0.0027	0.0741	0
DRB1*03:01	0.121	0.0707	0.0537	0.09	DRB1*12:08	0	0	0.0003	0
DRB1*03:02	0.001	0.0653	0	0.002	DRB1*13:01	0.065	0.0555	0.0238	0.043
DRB1*03:04	0.0001	0	0	0	DRB1*13:02	0.043	0.0645	0.0362	0.038
DRB1*03:05	0	0.0002	0	0	DRB1*13:03	0.013	0.037	0.0003	0.009
DRB1*03:06	0	0.0002	0	0	DRB1*13:04	0.0004	0.0131	0	0.001
DRB1*04:01	0.086	0.0229	0.0091	0.066	DRB1*13:05	0.001	0.0004	0	0.001
DRB1*04:02	0.011	0	0.0037	0.004	DRB1*13:06	0.0003	0	0	0
DRB1*04:03	0.008	0.0023	0.0345	0.011	DRB1*13:11	0	0.0002	0	0
DRB1*04:04	0.039	0.0069	0.0091	0.051	DRB1*13:12	0	0	0.0042	0
DRB1*04:05	0.007	0.0096	0.058	0.01	DRB1*13:16	0	0.0004	0	0
DRB1*04:06	0.001	0.0006	0.0206	0	DRB1*13:20	0	0.0002	0	0
DRB1*04:07	0.012	0.004	0.0014	0.049	DRB1*13:21	0.0001	0	0	0
DRB1*04:08	0.006	0.0006	0.0014	0.003	DRB1*13:31	0	0.0006	0	0
DRB1*04:09	0	0.0002	0	0	DRB1*13:36	0	0.0002	0	0
DRB1*04:10	0	0.0006	0.0037	0.002	DRB1*13:50	0	0	0.0003	0
DRB1*04:11	0.0003	0.0008	0	0	DRB1*14:01	0.025	0.0214	0.024	0.028
DRB1*04:13	0.0001	0	0	0	DRB1*14:02	0.001	0.0006	0.0003	0.063
DRB1*07:01	0.132	0.0977	0.082	0.101	DRB1*14:03	0.0001	0	0.0037	0
DRB1*07:03	0	0	0.0003	0	DRB1*14:04	0.0004	0.0004	0.0204	0.001
DRB1*08:01	0.025	0.0046	0.0045	0.016	DRB1*14:05	0.0001	0	0.0175	0
DRB1*08:02	0.002	0.001	0.013	0.016	DRB1*14:06	0.001	0	0.0028	0.003
DRB1*08:03	0.003	0.0004	0.0518	0.002	DRB1*14:07	0.001	0	0.0026	0
DRB1*08:04	0.002	0.0505	0	0.031	DRB1*14:08	0	0	0.0003	0
DRB1*08:05	0	0	0.0003	0	DRB1*14:12	0	0	0.0003	0
DRB1*08:06	0.0001	0.0052	0	0	DRB1*14:16	0.0001	0	0	0
DRB1*08:09	0	0	0.0023	0	DRB1*14:18	0	0	0.0014	0
DRB1*08:11	0.0003	0.001	0	0.007	DRB1*14:19	0	0	0.0006	0
DRB1*09:01	0.011	0.0316	0.1018	0.027	DRB1*14:22	0	0	0.0003	0
DRB1*10:01	0.007	0.0185	0.0311	0.007	DRB1*14:25	0	0	0.0003	0
DRB1*11:01	0.057	0.0871	0.0512	0.044	DRB1*15:01	0.135	0.0293	0.0792	0.104
DRB1*11:02	0.003	0.0391	0	0.004	DRB1*15:02	0.008	0.0017	0.0809	0.005
DRB1*11:03	0.005	0.0006	0	0.003	DRB1*15:03	0.001	0.1175	0.0006	0.003
DRB1*11:04	0.027	0.0056	0.0065	0.016	DRB1*15:04	0	0	0.0006	0
DRB1*11:06	0	0	0.0031	0	DRB1*15:06	0.0001	0	0.004	0
DRB1*11:08	0	0	0.0003	0	DRB1*15:07	0	0	0.0003	0
DRB1*11:10	0	0.0017	0	0	DRB1*16:01	0.012	0.001	0.0003	0.008
DRB1*11:11	0	0	0.0009	0	DRB1*16:02	0.003	0.0154	0.0195	0.029

HLA-DRB1 allele frequencies were obtained form http://www.allelefrequencies.net (25/05/2016) and correspond to: White (USA Colorado Univ Cord Blood Bank Caucasian, sample size: 3830); Black (USA African American pop 4, sample size: 2411); Asian (USA Asian pop 2, sample size: 1772) and N. A. Natives (USA NMDP North American Native Indian, sample size: 35791).
