# Supplementary material for: BCEPS: A Web Server to Predict Linear B Cell Epitopes with Enhanced Immunogenicity and Cross-Reactivity
Source: Cells. 2021 Oct 14;10(10):2744. doi: 10.3390/cells10102744 (PMC8534968; doi:10.3390/cells10102744)
Supplement: Supplementary file 1 [file cells-10-02744-s001.zip › Table_S2.pdf]

Table S2: B cell epitopes predicted in SARS-CoV-2 S protein

Start	*Epitopes*	*Acces*	*Flex*
202	KIYSKHTPINLVRDLPQG	0,17	0,25
243	ALHRSYLTPGDSSSGWTAGAAAYY	0,25	-0,16
398	DSFVIRGDEVRQIAPGQT	0,17	0,51
400	FVIRGDEVRQIAPGQTGKIADYNYKLPDDFTG	0,16	0,51
433	VIAWNSNNLDSKVGGNYNYLYRL	0,21	0,12
442	DSKVGGNYNYLYRLFRKS	0,15	0,54
446	GGNYNYLYRLFRKSNLKP	0,15	0,57
448	NYNYLYRLFRKSNLKPFERDISTE	0,16	0,67
456	FRKSNLKPFERDISTEIYQA	0,19	0,99
462	KPFERDISTEIYQAGSTP	0,21	0,91
492	LQSYGFQPTNGVGYQPYR	0,16	-0,07
556	NKKFLPFQQFGRDIADTTD	0,17	0,07
560	LPFQQFGRDIADTTDAVRDPQTLEILDITP	0,18	0,1
666	IGAGICASYQTQTNSPRRARSVASQSIIAYT	0,27	0,06
773	EQDKNTQEVFAQVKQIYKTPP	0,18	0,87
778	TQEVFAQVKQIYKTPPIKD	0,15	0,79
807	PDPSKPSKRSFIEDLLFNKV	0,19	0,75
1069	PAQEKNFTTAPAICHDGK	0,21	-0,03
1074	NFTTAPAICHDGKAHFPREG	0,19	-0,09
1101	HWFVTQRNFYEPQIITTD	0,2	0,17
1156	FKNHTSPDVDLGDISGINAS	0,25	0,94

Table summarizes the B cell epitopes predicted by BCEPS in SARS-CoV-2 surface glycoprotein S (YP_009724390.1). Predictions were obtained selecting a peptide size of 18 and the option to "Extend B-cell Epitopes". B cell epitopes located outside of the ectodomain or bearing n-glycosylation sites were sorted out.
